# Systematic Elucidation of the Mechanism of *Oroxylum indicum* via Network Pharmacology

**DOI:** 10.1155/2020/5354215

**Published:** 2020-07-14

**Authors:** Junmin Chen, Jianyong Chen, Jingrong Lu

**Affiliations:** ^1^Department of Otorhinolaryngology Head and Neck Surgery, Xinhua Hospital, Shanghai Jiaotong University School of Medicine, Shanghai 200092, China; ^2^Ear Institute, Shanghai Jiaotong University School of Medicine, Shanghai 200092, China; ^3^Shanghai Key Laboratory of Translational Medicine on Ear and Nose Diseases, Shanghai 200092, China

## Abstract

*Oroxylum indicum* (*O. indicum*) is an important traditional Chinese medicine that exerts a wide spectrum of pharmacological activities. However, the pharmacological effect of *O. indicum* and its mechanism of action have not to be systematically elucidated yet. In this study, the druggability for active compounds of *O. indicum* was assessed via Traditional Chinese Medicine Systems Pharmacology Database (TCMSP), and the potential drug targets of *O. indicum* were identified using PharmMapper database. Additionally, Gene Ontology (GO) and Kyoto Encyclopedia of Genes and Genomes (KEGG) pathway enrichment analyses were performed via WebGestalt. Drug-target-pathway networks were constructed using Cytoscape to give a visual view. Our findings revealed that *O. indicum* has extremely superb druggability with 41 putative identified target genes. GO, KEGG, and network analyses showed that these targets were associated with inflammatory immunoreactions, cancer, and other biological processes. In summary, *O. indicum* is predicted to target multiple genes/proteins and pathways that shape a network which can exert systematic pharmacological effects.

## 1. Introduction

Traditional Chinese medicine (TCM) is the most plentiful source of bioactive compounds or pharmaceutical components for drug development [1]. *Oroxylum indicum* (*O. indicum*), for instance, is an important herbal medicine that belongs to the Bignoniaceae family [2] and has been extensively used for centuries in many Asia countries [3]. It is known as Mu Hu Die [4] in China and also found throughout South and Southeast Asian countries such as Japan, Thailand, and India [5, 6]. *Oroxylum indicum* belongs to the Bignoniaceae family [2] and possesses a wide spectrum of pharmacological effects such as antioxidant [4], antimicrobial [7], antiallergic [8], anti-inflammatory [9], and anticancer [10–12] properties.

Attentions have been closely paid to *Oroxylum indicum* owing to its underlying roles in the prevention and clinical therapy of a wide spectrum of diseases, for instance, cough, asthma, pertussis, pharyngitis, chronic or acute bronchitis, arthritic and rheumatic problems, and tumors [2, 3, 12]. These findings suggest that *Oroxylum indicum* could be used as a valuable TCM by reason of the incision of complicated pathophysiological processes, identification of therapeutic targets, and discovery of potential molecular functions and involved pathways. However, the possible molecular mechanisms that *Oroxylum indicum* induces are rarely investigated. Simultaneously, utilization of computational approaches to identify the drug target molecules and uncover the hidden mechanisms is becoming the main current for the sake of saving time, money, and effort. Especially important, computational target identification and the following molecular mechanisms could speed up the progress of drug development.

**Figure 1:**
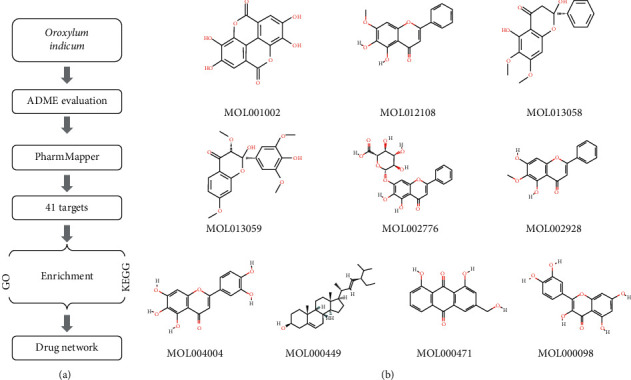
(a) Workflow for the identification of potential *Oroxylum indicum* target genes that integrates ADME evaluation, TCMSP, GO, and KEGG pathway analyses and network construction; (b) chemical structures for active compounds of *Oroxylum indicum* downloaded from TCMSP.

Therefore, we systematically investigated the pharmacological functions of *Oroxylum indicum* with the employment of computational approaches. Firstly, the active compounds of *Oroxylum indicum* were screened and evaluated using the Traditional Chinese Medicine Systems Pharmacology (TCMSP) resource. Next, the potential target genes of those active compounds were predicted by the PharmMapper database. Moreover, Gene Ontology (GO) and Kyoto Encyclopedia of Genes and Genomes (KEGG) pathway analyses were conducted with the utilization of the predicted targets. Finally, the drug-targets and targets-pathways pharmacological data of *Oroxylum indicum* were integrated and further used to construct a three-layer network. An overview of the analytical procedures for *Oroxylum indicum* target gene identification and mechanism investigation is illustrated in Figure 1(a).

## 2. Materials and Methods

### 2.1. Evaluation of Oral Bioavailability and Druglikeness by TCMSP

TCMSP (http://lsp.nwu.edu.cn/tcmsp.php) is a systematic database of pharmacology for natural compounds or TCM [13]. It provides information on the absorption, distribution, metabolism, and excretion (ADME) activities of compounds or TCMs, such as druglikeness (DL), oral bioavailability (OB), and blood-brain barrier (BBB) [14, 15].

Of all the ADME-related properties, DL and OB are the most vital features of administered drugs, due to their roles in evaluating the efficacy of the drug distribution to the circulatory system, and how drug-like a compound is with respect to factors like bioavailability. In the TCMSP database, DL was calculated based on the Tanimoto coefficient and molecular descriptors, while OB was evaluated on OBioavail 1.1 based on an in-house model [13, 14].

In this study, “*Oroxylum indicum*” was input to TCMSP database, and its pharmacokinetics activities were assessed at the molecular level. Compounds with OB ≥ 40% and DL ≥ 0.2 were screened for further investigation.

### 2.2. Target Identification via PharmMapper Database

PharmMapper (http://www.lilab-ecust.cn/pharmmapper/) is an online reverse-docking database for potential target identification of small molecules [16]. Given a small molecule in Mol2 or SDF format, it provides the top 300 targets sorted by the normalized fit score in descending order. In the present study, the Mol2 format files of the active compounds identified were downloaded from TCMSP and uploaded individually to the PharmMapper. Only human protein targets were chosen and other arguments were set to default values. The top 10 targets identified of the individual compound were selected for further investigation.

### 2.3. GeneMANIA Analysis

GeneMANIA (http://www.genemania.org) is an online tool for generating hypotheses concerning gene function, analyzing gene sets, and prioritizing genes for functional assays [17].

Given a query gene list, GeneMANIA can discover functionally similar genes based on curated genomics and proteomics data. After selecting *Homo sapiens* from the organism option, the target gene list of interest was input to the search box, and the results were further collated.

### 2.4. Gene Function and Pathway Enrichment Analyses

We employed a Web-based gene set analysis toolkit (WebGestalt, http://www.webgestalt.org/option.php) to systematically investigate the functions and pathway enrichment information on the target genes we predicted [18]. The gene of interest was entered into WebGestalt web server utilizing overrepresentation enrichment analysis (ORA) approach with Gene Oncology [19] and KEGG databases [20, 21] and other default parameters. False discovery rate (FDR) adjusted *p* value less than 0.05 was considered to be significant statistically.

### 2.5. Network Construction

For comprehensively understanding the complex association among the drug, target gene, and related pathways, we used Cytoscape (v 3.7.2) to build and analyze the drug-targets-pathways networks.

## 3. Results

### 3.1. Screening of Active Compounds from TCMSP

TCMSP provides detailed information on vital ADME-related activities such as human oral bioavailability (OB) and druglikeness (DL). The active compounds of *Oroxylum indicum* were identified based on the ADME-related properties from the TCMSP database, with the threshold OB ≥ 40% and DL ≥ 0.2. 10 active compounds were yielded and finally chosen for further investigation (Figure 1(b) and Table 1).

### 3.2. Identification of Potential Drug Targets

Potential targets for the 10 active compounds of *Oroxylum indicum* were identified using PharmMapper [16] with selecting the top 10 targets of individual compound based on the normalized fit score. We obtained their official gene symbol and gene ID from PDB and UniProt and the Gene database of the National Center for Biotechnology Information (NCBI), yielding 41 targets after the removal of duplicates. After the removal of the duplicates, we finally identified 41 unique target genes of the 10 active compounds (Table 2). These 41 identified target genes were utilized for further investigation.

### 3.3. GeneMANIA Analysis

**Figure 2:**
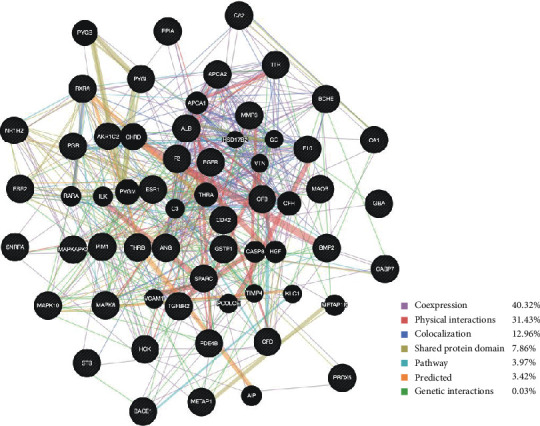
Protein network of target genes. Black nodes represent target proteins, and connecting colors indicate different correlations. Functional associations between targets were investigated using GeneMANIA. Genes in black circles were query terms while these in grey circles indicate genes associated with query genes.

Among the 41 target genes and their interacting genes, it was uncovered that 40.32% displayed coexpression characteristics, 31.43% had physical interactions, and 12.96% engaged in colocalization. Other results, including shared protein domains, pathway, and genetic interactions, are illustrated in Figure 2.

### 3.4. GO and KEGG Pathway Analyses

**Figure 3:**
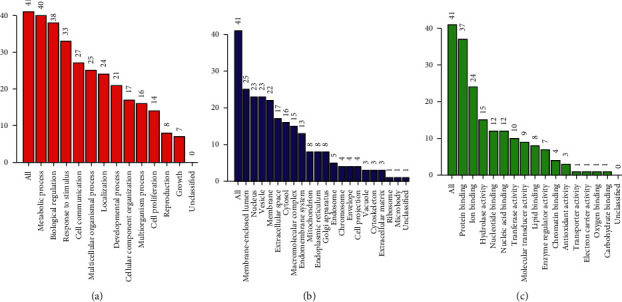
GO map of putative target genes. (a) Biological process categories. (b) Cellular component categories. (c) Molecular function categories.

**Figure 4:**
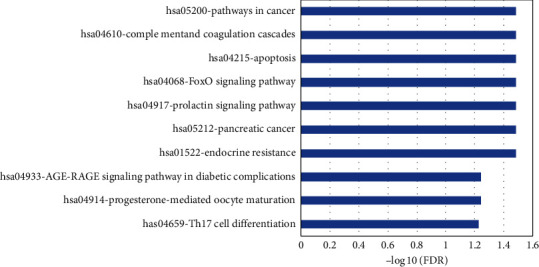
KEGG pathway analysis of putative target genes. *X*-axis represents negative log 10 of false discovery rate (FDR) adjusted *p* value while *y*-axis demonstrates the enriched KEGG pathways with ID and name.

To further understanding the 41 predicted targets, GO and KEGG enrichment analyses were performed via applying WebGestalt. As demonstrated in Figure 3, the top seven functions were metabolic process (40/41), biological regulation (38/41), protein binding (37/41), response to stimulus (33/41), cell communication (27/41), multicellular organismal process (25/41), and membrane-enclosed lumen (25/41). These GO terms were highly relevant to anti-inflammatory activities, particularly for pharyngitis, chronic or acute bronchitis, and other respiratory diseases, As for the pathway analysis, we found the 41 target genes participated in 10 KEGG pathways with significant FDR adjusted *p* value including pathways in cancer, complement and coagulation cascades, and apoptosis (Figure 4).

### 3.5. Network Analysis

**Figure 5:**
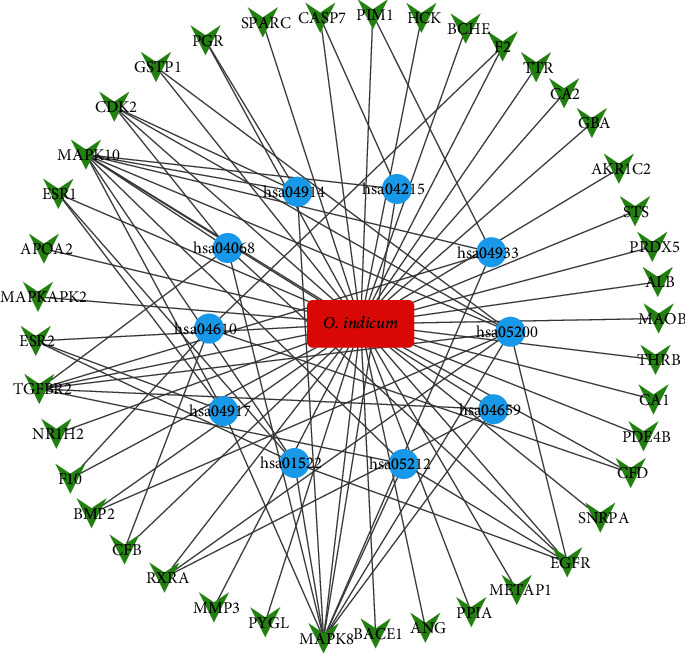
*O. indicum*-target-pathway network. The red oblong, green invert triangles, and blue circles indicate drug, target genes, and related pathways, respectively.

Based on target identification and pathway analysis, an entire drug, targets, and involved pathways network was built via Cytoscape (v 3.7.2). As demonstrated in Figure 5, this three-layer network had 52 nodes and 86 edges. The red oblong, green invert triangles, and blue circles represent drug, target genes, and related pathways, respectively.

## 4. Discussion

Traditional Chinese medicine (TCM) has been applied extensively to prevent and treat various kinds of diseases owing to its high efficiency, no drug resistance, and low toxicity [22, 23]. Thus, the development of active compounds derived from TCM on the drug design and discovery process should be prioritized urgently [24–27]. *In silico* analyses can improve pharmacokinetic modeling, prediction, as much as toxicity, and metabolic endpoints; all of which streamline and speed up the drug development progress [28, 29].

Identification of target genes is the first step in drug discovery. It has been revealed that more and more active drugs or compounds interact with multiple genes or proteins to exert their pharmacological functions [30–33]. Different kinds of *in silico* target identification approaches have been developed and are widely used towards this end. As shown in Table 2, 41 potential target genes of *Oroxylum indicum* were identified using computational approaches. GeneMANIA analysis, with information on coexpression, physical interactions, and colocalization, as well as pathway, shared protein domains, and genetic interactions, indicated that these target genes and their interacting genes may have identical or similar functions.

Accordingly, we identified an inflammatory role for *Oroxylum indicum* in pharyngitis and chronic or acute bronchitis. For example, the majority of the identified targets of *Oroxylum indicum* were enriched in biological regulation, response to stimulus, cell communication, and so on. These were all essential for *Oroxylum indicum* to exert its inflammatory role. Similarly, Begum et al. also found *Oroxylum indicum* plays a vital role in inhibiting inflammation, antiulcerative, enhancing immunity, and lowering blood glucose [6]. These findings coincide closely with our results via GO and KEGG analyses. Furthermore, we discovered that some of the predicted target genes of *Oroxylum indicum* were associated with cancer, such as EGFR, PIM1, ESR1/2, and MAPK8/10. These genes are closely related to cell proliferation, differentiation, or migration and participant in pathways in cancer, complement and coagulation cascades, apoptosis, signaling pathway, and so on. More importantly, some of these are transcription factors that coregulate several pathways simultaneously, like EGFR and ESR1/2. Consistently, Li et al. have also discovered that *O. indicum* induces apoptosis via PI3K/Akt/PTEN signaling pathway in liver cancer [12]. These interesting findings may elucidate the potential mechanism of antitumor role that *Oroxylum indicum* exerts.

The drug-targets network illustrated in Figure 5 also demonstrated that *Oroxylum indicum* has multiple targets to exert multiple pharmacological effects. Multiple target therapeutic drugs are more effective for the treatment of complex diseases, such as cough, asthma, pertussis, and cancers, and are less vulnerable to drug resistance. Therefore, *Oroxylum indicum* could be a promising resource that may be used as a lead compound, chemical moiety, or active ingredients for further drug development. Nevertheless, we have to acknowledge that there are some limits and bias in our analyses due to the databases we employed, for example, the release, update date, and resources they used.

## 5. Conclusion

In short, we would like to emphasize that *Oroxylum indicum* is a valuable TCM and contains quite promising compounds for the development of an effective and safe multitargeted anti-inflammatory and antitumor medicament. This study provides novel insight into the perspectives and challenges as for the *Oroxylum indicum* research and clinical application in future investigations.

## Figures and Tables

**Table 1:** Pharmachological properties for active compounds of *Oroxylum indicum*.

ID	OB (%)	DL	Molecule name
MOL001002	43.06	0.43	Ellagic acid
MOL012108	41.16	0.23	Negletein
MOL013058	41.52	0.28	2,5-Dihydroxy-6,7-dimethoxyflavone
MOL013059	53.26	0.42	3,7,3′,5′-Tetramethoxy-2-hydrochroxyflavone
MOL002776	40.12	0.75	Baicalin
MOL002928	41.37	0.23	Oroxylin A
MOL004004	46.93	0.28	6-OH-Luteolin
MOL000449	43.83	0.76	Stigmasterol
MOL000471	83.38	0.24	Aloe-emodin
MOL000098	46.43	0.28	Quercetin

**Table 2:** Drug targets for active compounds of *Oroxylum indicum*.

No.	Gene ID	Gene symbol	Target name
1	5292	PIM1	Proto-oncogene serine/threonine-protein kinase Pim-1
2	3055	HCK	Tyrosine-protein kinase HCK
3	2099	ESR1	Estrogen receptor
4	590	BCHE	Cholinesterase
5	2147	F2	Prothrombin
6	5241	PGR	Progesterone receptor
7	1956	EGFR	Epidermal growth factor receptor
8	7276	TTR	Transthyretin
9	760	CA2	Carbonic anhydrase 2
10	2629	GBA	Glucosylceramidase
11	1646	AKR1C2	Aldo-keto reductase family 1 member C2
12	9261	MAPKAPK2	MAP kinase-activated protein kinase 2
13	650	BMP2	Bone morphogenetic protein 2
14	412	STS	Steryl-sulfatase
15	213	ALB	Serum albumin
16	4129	MAOB	Amine oxidase [flavin-containing] B
17	336	APOA2	Apolipoprotein A-II
18	7068	THRB	Thyroid hormone receptor-beta
19	840	CASP7	Caspase-7
20	4314	MMP3	Stromelysin-1
21	5599	MAPK8	Mitogen-activated protein kinase 8
22	5602	MAPK10	Mitogen-activated protein kinase 10
23	759	CA1	Carbonic anhydrase 1
24	1017	CDK2	Cell division protein kinase 2
25	2950	GSTP1	Glutathione S-transferase P
26	5142	PDE4B	cAMP-specific 3,5-cyclic phosphodiesterase 4B
27	1675	CFD	Complement factor D
28	6256	RXRA	Peroxisome proliferator-activated receptor-gamma
29	7376	NR1H2	Oxysterols receptor LXR-beta
30	6626	SNRPA	U1 small nuclear ribonucleoprotein A
31	2159	F10	Coagulation factor X
32	25824	PRDX5	Peroxiredoxin-5, mitochondrial
33	23173	METAP1	Methionine aminopeptidase 1
34	5478	PPIA	Peptidyl-prolyl cis-trans isomerase A
35	283	ANG	Angiogenin
36	2100	ESR2	Estrogen receptor-beta
37	23621	BACE1	Beta-secretase 1
38	6678	SPARC	SPARC
39	7048	TGFBR2	TGF-beta receptor type-2
40	5836	PYGL	Glycogen phosphorylase, liver form
41	629	CFB	Complement factor B

## Data Availability

The data used to support the findings of this study are available from the corresponding author upon request.
